# Risk of ischemic stroke in patients with hypertrophic cardiomyopathy in the absence of atrial fibrillation – a nationwide cohort study

**DOI:** 10.18632/aging.102532

**Published:** 2019-12-02

**Authors:** Ting-Tse Lin, Yen-Ling Sung, Tsung-Yu Ko, Chih-Kuo Lee, Lian-Yu Lin, Jimmy Ji-Ming Juang, Cho-Kai Wu

**Affiliations:** 1 Department of Internal Medicine, National Taiwan University Hospital Hsin-Chu Branch, Hsinchu, Taiwan; 2 Department of Biological Science and Technology, National Chiao Tung University, Hsinchu, Taiwan; 3 College of Medicine, National Taiwan University, Taipei, Taiwan; 4 Department of Electrical and Computer Engineering, National Chiao Tung University, Hsinchu, Taiwan; 5 Institute of Biomedical Engineering, College of Electrical and Computer Engineering, National Chiao Tung University, Hsinchu, Taiwan; 6 Division of Cardiology, Department of Internal Medicine, National Taiwan University College of Medicine and Hospital, Taipei, Taiwan

**Keywords:** hypertrophic cardiomyopathy, risk of ischemic stroke, age

## Abstract

Ischemic stroke (IS) is a catastrophic complication of hypertrophic cardiomyopathy (HCM) with aging. We investigated the incidence of IS in HCM patients without atrial fibrillation (AF) and compared the relative risk of IS with matched general population with AF. This study identified 17,371 HCM patients without AF and utilized propensity-score-matching to identify one-to-one matched control of general population with AF receiving oral anti-coagulants (OACs). During a median follow-up of 7.3 years, 847 (4.9%) subjects experienced IS with the incidence of 0.589/100 person-years. The corresponding matched controls experienced 788 (4.5%) events with the incidence of 0.494/100 person-years. Compared with control, HCM patients had similar risk of IS (Hazard ratios [HRs] 0.965, 95% confidence interval [CI] 0.854-1.091). HCM patients with age above 65 years had a significantly increased risk of IS (age 65-74 years, HR 1.278, 95% CI 1.070-1.335; age ≥75 years, HR 1.757, 95% CI 1.435-2.152). Stratified by CHA_2_DS_2_-VASc score, HCM subjects with score 0, 1 and 2 had significantly increased risk of IS than control while those with score ≥2 had similar risk as control. Compared with general population with AF, HCM patients without AF had similar risk of IS, suggesting OACs might be necessary in HCM patient without AF.

## INTRODUCTION

HCM is one of the most common genetic cardiac disorders with markedly heterogeneous clinical manifestations and complications, including ischemic stroke and systemic embolism [1, 2]. Since patients with hypertrophic cardiomyopathy (HCM) and atrial fibrillation (AF) have high risk of stroke, it is recommended that all patients with AF should receive treatment with oral anticoagulants (OACs) regardless of CHA_2_DS_2_-VASc score. For patients who are unable to take OACs, dual anti-platelets could be considered [1]. However, AF is not the only predictor of the thrombo-embolic events in patients with HCM. Older age and left atrium dilation are also significantly associated with the occurrence of ischemic stroke and systemic emboli [3, 4]. Since the AF of patients with HCM is often paroxysmal and asymptomatic, the diagnosis is difficult and the silent and subclinical paroxysmal episode of AF may lead to stroke in patients without previously documented AF [5, 6]. Therefore, it is recommended that patients with HCM and left atrial diameter ≥45 mms should undergo 6–12 monthly 48-hour ambulatory ECG monitoring to detect AF [1]. On the other hand, age is an important determinant in the natural history of HCM and we previously recognized HCM is a progressive disease. Nevertheless, aging in HCM may represent a negative risk marker for sudden death and HCM patients of advanced age (> 60 years) are more likely to die for non-cardiac competing morbidities [7]. Therefore, the risk of ischemic stroke and epidemiology in different age classification groups of patients with HCM in the absence of AF is an important issue and the nature is unknown. The aim of our study was to investigate the risk of ischemic stroke and clinical features of HCM patients without documented AF in different age classification.

## RESULTS

### Patient characteristics

There were 17,371 patients who met the study inclusion criteria and 17,371 matched AF subjects without HCM treated as the reference group. The median follow-up time was 7.3 years. Clinical and demographic characteristics were listed in Table 1 and there was no significant difference between two groups. The mean age was 61 years and around 45% subjects were women. Nearly 70% subjects had hypertension, 25% had diabetes and 40% had hyperlipidemia. However, there were significantly more prescriptions of antiplatelet and ACEI/ARB in HCM patients than those of reference group.

**Table 1:** Baseline characteristics of hypertrophic cardiomyopathy patients without atrial fibrillation and the matched general population with atrial fibrillation.

**Characteristics**	**HCM patients without AF**	**Matched patients with AF**	**P value**
N	17371	17371	
**Matched variables**			
Female, n (%)	8183 (47.1)	7780 (44.8)	0.102
Age, years	61±15	62±13	0.291
CHA_2_DS_2_-VASc score	3.09±1.61	3.00±1.70	0.562
Hypertension, n (%)	12100 (69.7)	11695 (67.3)	0.167
Diabetes	4729 (26.2)	4035 (23.2)	0.089
Coronary artery disease	9704 (55.9)	9910 (57)	0.276
Hyperlipidemia	6813 (39.2)	6618 (38.1)	0.214
Hospitalization for heart failure	4865 (28)	5055 (29.1)	0.312
Chronic kidney disease	1581 (9.1)	1492 (8.5)	0.297
**Other variables**			
Antiplatelet	9938 (57.2)	7942 (45.7)	0.002
ACEI/ARB	11022 (63.5)	9872 (56.8)	0.001
Beta-blockers	7371 (42.4)	7172 (41.3)	0.236
Calcium channel blockers	7371 (42.4)	8192 (47.1)	0.067
Statins	5633 (32.4)	5849 (33.6)	0.179

CHA2DS2-VASc indicates congestive heart failure, hypertension, age ≥75 (doubled), diabetes mellitus, prior stroke or transient ischemic attack (doubled), vascular disease, age 65–74, female.

Abbreviations: ACEI, angiotensin-converting enzyme; ARB, angiotensin II receptor blocker.

### Outcome of ischemic stroke

**Figure 1:**
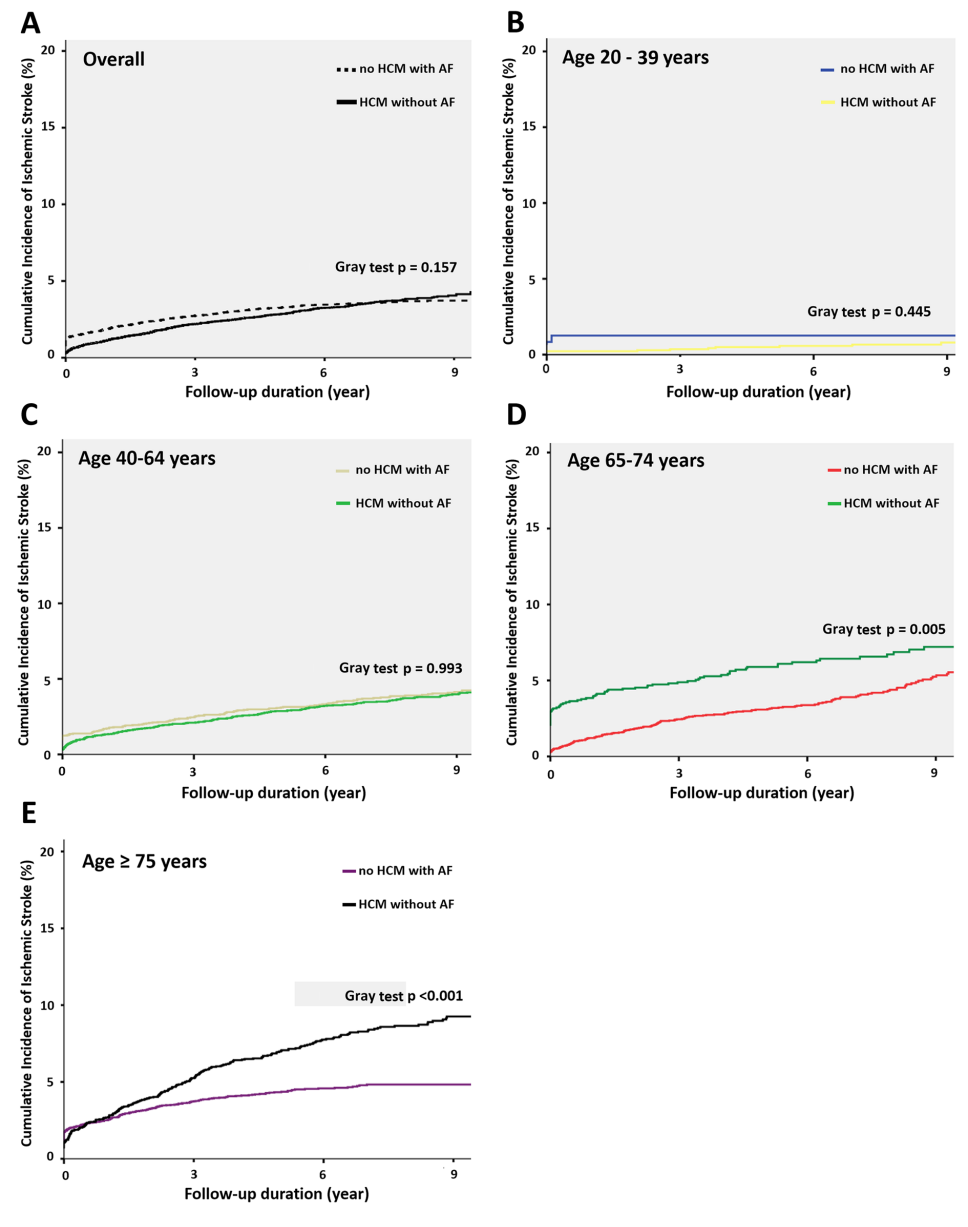
**Cumulative incidence function curves for ischemic stroke in different age groups.** (**A**) Survival curve with the Fine and Gray test describing ischemic stroke among patients with HCM without AF and matched general population with AF. The Fine and Gray test showed no significant difference (P = 0.157). (**B**) Survival curve in the subgroup with age of 20-39 years. The Fine and Gray test showed no significant difference. (p=0.445). (**C**) Survival curve in the subgroup with age of 40-64 years. The Fine and Gray test showed no significant difference. (p=0.993). (**D**) Survival curve in the subgroup with age of 65-74 years. The Fine and Gray test showed significant difference. (p=0.005). (**E**) Survival curve in the subgroup with age above 75 years. The Fine and Gray test showed significant difference. (p < 0.001). Abbreviations: AF, atrial fibrillation; HCM, hypertrophic cardiomyopathy.

The overall incidence of ischemic stroke was 4.9% of HCM patients without AF and 4.5% of reference group during a median follow-up period of 7.3 years (25–75%, 5.2–9.3 years). Predictors of IS in patients with HCM without documented atrial fibrillation were age, CHA_2_DS_2_-VASc and sudden cardiac death in multivariate analysis model (Supplementary Table 1). In both groups, we classified them into 4 different subgroups of age between 20-39, 40-64, 65-74 and above 75 years. We observed progressive increased annual ischemic stroke rate (100-person year) in HCM subjects (20-40 years, 0.167, 95% CI 0.079-0.208; 40-65 years, 0.372, 95% CI 0.271-0.398; 65-75 years, 0.743, 95% CI 0.687-0.792 and >75 years, 1.048, 95% CI 0.942-1.216) (Table 2). There was a similar trend of annual risk of ischemic stroke in matched reference group (20-40 years, 0.114, 95% CI 0.075-0.213; 40-65 years, 0.389, 95% CI 0.285-0.456; 65-75 years, 0.575, 95% CI 0.432-0.684 and >75 years, 0.528, 95% CI 0.473-0.625). Overall, treated the general population with AF as reference group, the relative risk of ischemic stroke for HCM patients was comparable (HR 0.965, 95% CI 0.854 – 1.091, p=0.215). However, the relative risk of ischemic stroke in different age groups showed continuously increased from younger to older age group strata. There was no difference of risk of ischemic stroke among subjects with age 20-40 years (HR 0.595, 95% CI 0.151 – 2.346, p = 0.712), and 40 – 65 years (HR 0.642, 95% CI 0.151 – 2.346, p = 0.712). By contrast, there was an significantly increased risk of ischemic stroke for HCM subjects with age 65 – 75 years (HR 1.278, 95% CI 1.070 – 1.335, p = 0.025) and > 75 years (HR 1.757, 95% CI 1.435 – 2.152, p = 0.012) when compared with matched general population with AF. The results of subdistribution Cox proportional model also demonstrated that HCM patients without AF in the older age group were associated with a higher risk of ischemic stroke during the follow-up period than the matched controls (Supplementary Table 2). In the sensitivity analyses, we stratified our cohort into 6 subgroups on the basis of age with the same interval of 10 years (age 25–34, 35–44, 45–54, 55–64, 65–74 and > 75 years). The major finding remained unchanged in the sensitivity analysis (Supplementary Table 3). The Kaplan-Meier survival curves were illustrated in Figure 1. The overall cumulative incidence curves showed no significant different between HCM patients without AF and matched controls (log-rank test, p = 0.157) (Figure 1A). However, the cumulative incidence curve with the log-rank test demonstrated that patients in the older age subgroup were associated with a higher risk of ischemic stroke during the follow-up period (age 20–44 years, p=0.445; age 45-64 years, p=0.993; age 65–74 years, p=0.005 and age ≥ 75 years, p<0.001) (Figure 1B–1E).

**Table 2:** Annual ischemic stroke rate of patients with hypertrophic cardiomyopathy in the absence AF and matched control group stratified by age.

	**Number of subjects**	**Number of event**	**Person-year**	Annual stroke rate (95% CI)*, 100-person year
**HCM in the absence AF**				
Overall	17371	847	143635	0.589 (0.494-0.629)
Age, years				
20-39 y/o	1762	24	14363	0.167 (0.079-0.208)
40-64 y/o	7586	229	61474	0.372 (0.271-0.398)
65-74 y/o	4353	284	38231	0.743 (0.687-0.792)
≥ 75 y/o	3670	310	29567	1.048 (0.942-1.216)
**Matched general population with AF**		
Overall	17371	788	159263	0.494 (0.385-0.513)
Age, years				
20-39	389	5	4271	0.114 (0.075-0.213)
40-64	3955	153	39452	0.389 (0.285-0.456)
65-74	4128	235	40911	0.575 (0.432-0.684)
≥ 75	8899	395	74629	0.528 (0.473-0.625)

*Incidence rates of ischemic stroke were calculated by dividing the number of events by person-time at risk, with the 95% confidence interval (CI) estimated by exact binomial probabilities

Abbreviations: AF, atrial fibrillation; CI, confidence interval; HCM, hypertrophic cardiomyopathy.

**Table 3:** Hazard ratio (95% confidence interval) of ischemic stroke in patients with hypertrophic cardiomyopathy but in the absence of atrial fibrillation, treated the matched general population with atrial fibrillation as reference group.

**HCM w/o AF vs General population with AF**	**Hazard ratio ƚ**		
	**General population**	**HCM patients**	**95% confidence interval**
Overall	1	0.965	0.854-1.091
Age, years			
20-39	1	0.595	0.151-2.346
40-64	1	0.642	0.497-1.829
65-74	1	1.278	1.070-1.335*
≥ 75	1	1.757	1.435-2.152*

* p value < 0.05.

ƚ The relative risk was calculated by Cox proportional hazard model and adjusted for age, gender, risk factors (hypertension, diabetes mellitus and hyperlipidemia), comorbidities (coronary artery disease, chronic kidney disease, hospitalization for heart failure), and medication usage.

Abbreviations: AF, atrial fibrillation; HCM, hypertrophic cardiomyopathy.

**Figure 2:**
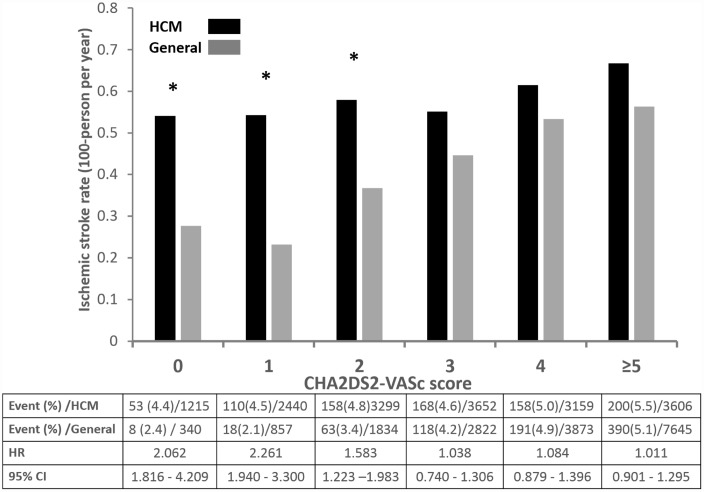
**Annual risk of ischemic stroke in patients with HCM without AF and matched general population without AF.**

We also stratified our cohort according to CHA_2_DS_2_-VASc score into 5 groups (score 0, 1, 2, 3, 4 and ≥ 5). Compared with matched general population, HCM subjects had significantly increased risk of ischemic stroke in those with CHA2DS2-VASc score ≤ 2 (score 0, HR 2.062, 95% CI 1.816 – 4.209, p value 0.026; score 1, HR 2.261, 95% CI 1.940 – 3.300, p value 0.018; score 2, HR 1.383, 95% CI 1.023 – 2.201, p value 0.034) (Figure 2). However, those with score ≥ 3 had similar risk of ischemic stroke compared with control.

## DISCUSSIONS

In this study, we investigated and compared the risk of ischemic stroke in HCM patients without AF and the matched general cohort with AF. We found that both groups overall had comparable risks of ischemic stroke after 7-year follow-up. Stratified by CHA_2_DS_2_-VASc score, there was a similar risk of ischemic stroke between two groups. However, after stratified by age, patients with HCM and age above 65 years, the risk of stroke was significantly increased compared with matched general population. Our findings suggest HCM itself serves the similar risk of ischemic stroke as AF. Furthermore, elder age but not CHA2DS2-VASc score is highly associated with increased risk of ischemic stroke in HCM patients in the absence of AF.

Owing to the pathophysiology of HCM, AF is the most common arrhythmia in patients with HCM, whose the prevalence and annual incidence of thromboembolism events were 27.1% and 3.8% respectively [1, 8]. However, few data are available on the occurrence and profile of these events in these patients without AF. Haruki et al. had reported the incidence of stroke was about 1.0 % per year in the HCM cohort without documented AF before enrollment. However, around 40% of subjects experienced AF before incidence of stroke or systemic emboli. Our cohort excluded HCM subjects with diagnosis of AF at outpatient visit or discharge during whole follow-up period and the incidence of only ischemic stroke was 0.6% per year. Along with increased age, the higher incidence of ischemic stroke was observed in our cohort. These findings were consistent with previous studies [1, 4, 9–11]. Furthermore, we constructed a matched cohort of general population with AF as reference. In the HCM cohort without AF, subjects had similar risk of ischemic stroke as reference cohort, implying HCM might be a profound thromboembolic risk as AF [10]. Aging and other cardiovascular disease cause atrial cardiomyopathy, that can result in AF and thromboembolism. The abnormal atrial substrate could promote thrombosis in the absence of AF. Once AF develops, the dysrhythmia causes contractile dysfunction and stasis, which further increases the risk of thromboembolism [12]. In patients with HCM, LA enlargement could be considered as an adaptive response for systolic anterior movement-related MR and elevated left ventricular filling pressure [13, 14]. Given AF is the most important surrogate marker and the final rhythm of the progression of atrial cardiomyopathy, there was a high prevalence of incident AF in HCM patients. In HCM patients with normal LA size, LA strain showed good predictive value of new-onset AF, suggesting abnormal atrial substrate preceding the development of AF [15]. The abnormal LA substrate and function in HCM patients without documented AF could increase the risk of thromboembolism and even sudden cardiac death [16]. Therefore, in our cohort whose HCM patients without AF, the risk of ischemic stroke was not different from general populations with AF receiving NOACs. Furthermore, we noted CHA2DS2VASc scores was associated with ischemic stroke in the HCM group without AF (OR: 1.102; 95% CI = 1.036 – 1.154). A prospective study found that CHADS2 score correlated with higher left atrial fibrosis, and an association has been noted between inflammatory parameters and CHA2DS2-VASc score in patients with AF [17, 18]. Atrial fibrosis and associated inflammation caused by HCM may contribute to thromboembolism and perhaps explain why CHA2DS2-VASc score could predict the ischemic stroke in patients without AF.Of note, the rate of ischemic stroke was significantly higher in HCM patients with lower CHA_2_DS_2_VASc scores (score ≤2). Our findings suggest HCM itself is a strong clinical risk of atrial thromboembolism and might be recognized and treated as AF.

In our study, CHA2DS2-VASc score, age and occurrence of sudden cardiac death were significantly associated with incident ischemic stroke. Furthermore, HCM subjects with age above 65 years had increased risk of ischemic stroke when compared with reference group. It is usually conceived that achieving older age in HCM may itself convey relatively protection from sudden death and afford more favorable prognosis. This principle is underscored by the relatively low sudden death events [19]. However, the most common events is embolic stroke in the elder HCM patients, underscoring anti-coagulation therapy early in those patients [7]. Our findings support that considering anticoagulation therapy in elder HCM patients without atrial fibrillation could prevent further embolic events.

### Limitations

There are several limitations to this retrospective cohort database study. First, baseline comorbidities and estimation of incidence of ischemic stroke were on diagnostic codes registered by the physician. Although administrative databases are increasingly used for clinical research and high concordance between claims records in the NHIRD and patient self-reports at surveys, our study is still potentially susceptible to errors arising from not completely coding all disease. However, the large size of the database in this study should be sufficient to reach an accurate statistical conclusion [20]. Second, the conventional risk of AF and ischemic stroke in HCM patients was not recorded, such as left ventricle wall thickness, left atrial diameter and continuous electrocardiogram monitoring [3]. Therefore, we cannot investigate the impact of these parameters on the incidence of stroke. Third, given the conceived better prognosis of elder HCM patients, the physician would less aggressively investigate HCM-related diseases. As a result, AF might be under-diagnosed in clinical practice. Fourth, our study enrolled only Taiwanese participants, and we do not know whether our result could be extrapolated to non-Asian populations; therefore, further study should be conducted.

## MATERIALS AND METHODS

### Source of data

This large-scale, longitudinal cohort study used integrated medical and pharmacy claims data from National Health Insurance Research Database (NHIRD) in Taiwan. The National Health Insurance program has provided compulsory universal health insurance in Taiwan since 1995. More than 98% of the total Taiwanese population of 23 million is covered by the program [21]. The NHIRD contains nearly complete claims history of diagnosis and procedures, provided as the International Classification of Diseases Ninth Revision Clinical Modification (ICD-9-CM) codes, and drug dispensing for every beneficiary. The Bureau of National Health Insurance performs routine validations of the diagnoses by reviewing the original medical charts of all of the patients who applied for catastrophic illness registration. To comply with data privacy regulations, personal identities were encrypted and all data were analyzed in a de-identified manner. The protocol for this study was approved by the Institutional Review Board of National Taiwan University Hospital.

### Study population and outcomes

**Figure 3:**
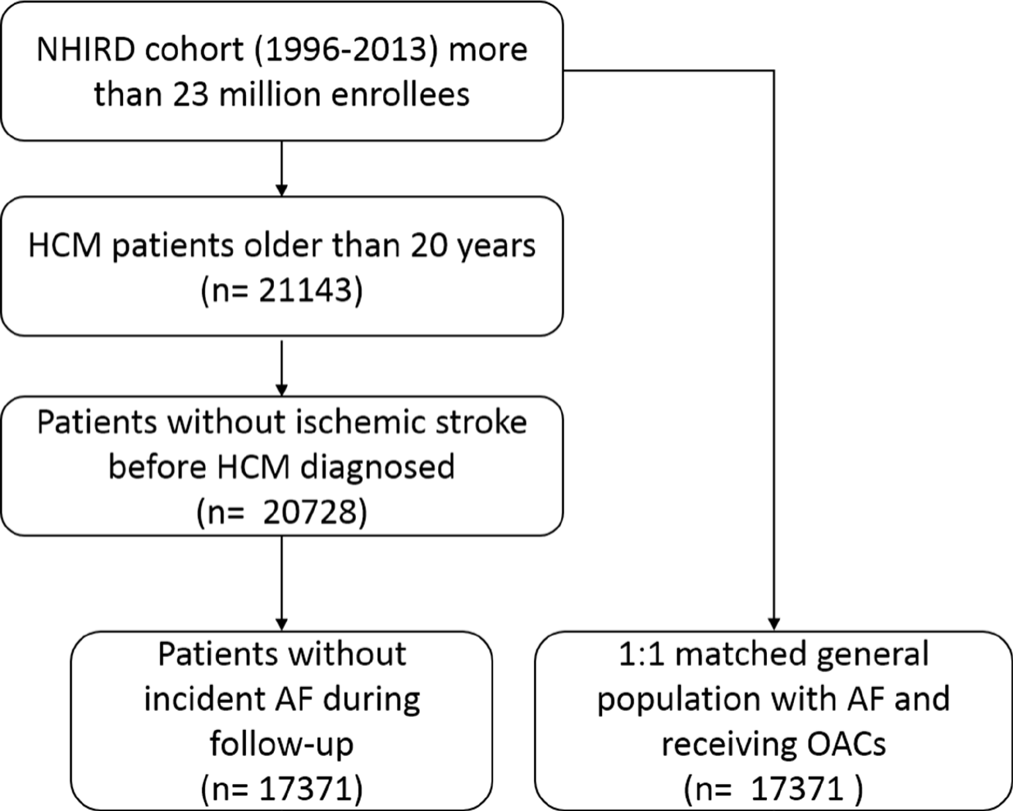
**Patient flow diagram.** Abbreviations: AF, atrial fibrillation; HCM, hypertrophic cardiomyopathy; NHIRD, National health insurance research database.

We investigated the database of NHIRD during year of 1996 to 2013. The index date for the study cohort was identified as the date of the first-time that had a diagnosis of HCM (ICD9-CM: 425.1). We identified all patients who were above 20 years old. The exclusion criteria included the following: (1) prior diagnosis of ischemic stroke (ICD9-CM code: 434, 434.1, 435, 436, 437, 434.91) before index date, (2) ambulatory visit for AF (ICD9-CM code, 427.3) during follow-up (Figure 3). All subjects were then followed from the index date to the date of first ischemic stroke event, or Dec. 31, 2013 if no event occurred with a median follow-up duration of 7.3 years (25–75%, 5.2–9.3 years). Comorbidity was defined by diagnoses at hospital discharge or in clinic records. For our study population, we searched the database to see if they had hypertension (ICD-9-CM codes: 401.X-405.X), diabetes mellitus (250.X, 249.X), hyperlipidemia (272.X), coronary artery disease (ICD9-CM code, 411.X-414.X, V17.3, V81.0), heart failure hospitalization (ICD9-CM code, 428.0-428.3, 429.9), chronic kidney disease (ICD9-CM code, 585.X-588.X). Medications that were dispensed at time of index date, including antiplatelet, angiotensin converting enzyme inhibitors (ACEIs), angiotensin receptor blockers (ARBs), beta-blockers, calcium channel blockers (CCB) and statins were identified.

The primary endpoint was diagnosis of ischemic stroke, based on ICD-9-CM coding (ICD9-CM code, 427.3, 435.X, A299) in any ambulatory visit and discharge diagnoses. The main purpose of the study was to investigate the risk of long-term development of ischemic stroke of HCM patients in the absence of AF. Therefore, we used latent class models to identify matched subjects without HCM but with AF from database of NHIRD sharing a similar underlying age, sex, and comorbidities including hypertension, diabetes, hyperlipidemia and chronic kidney disease. Of note, we excluded subjects not receiving oral anti-coagulants during follow-up. The index date of matched subjects was defined as the diagnosis of AF. Each HCM patient and his/her corresponding matched control had the same date of the diagnosis of HCM/AF. Our study design with the same index date of both groups was similar as new user (incident user) designs, preventing the problem of depletion of susceptibles. Subjects in our study were classified into two groups of patients with HCM in the absence of AF and matched subjects with AF.

### Statistical analyses

For comparison of the baseline characteristics between two groups, paired t-test (continuous variables) and the chi-squared (categorical covariates) test was performed. To reduce the potential bias and to make the two groups more comparable, the propensity score was applied. We derived a propensity score, which is the logit (probability) for receiving beta-blockers treatment from a logistic regression model by using matching variables listed in Table 1 (age, sex, CHA2DS2-VASc score, hypertension, diabetes, coronary artery disease, hyperlipidemia, hospitalization for heart failure and chronic kidney disease). The predicted accuracy of the logistic model was assessed with an area under the receiver operating characteristic curve (C statistic), which was 0.824 (95% CI 0.792 – 0.846). According to the propensity score, patients were selected by 1:1 matching without replacement using the nearest neighbor method. A caliper width of 0.15 standard deviations (SDs) was used for matching as our previous studies [21, 22]. The annual risk of ischemic stroke was calculated for patients who were stratified into 4 groups on the basis of age (20 to 39 years, 40 to 64 years, 65 to 75 years, and above 75 years). For survival analyses, multivariate Cox’s proportional hazard regression analyses were used to derive the adjusted HR for developing ischemic stroke by using matched population with AF as controls [23]. The model was adjusted for age, gender, risk factors (hypertension, diabetes mellitus and hyperlipidemia), comorbidities (coronary artery disease, chronic kidney disease, hospitalization for heart failure), and medication usage. On the other hand, considering that all-cause death is a competing risk for ischemic stroke in HCM population, we also estimated the association between each HCM patient and incidence of ischemic stroke during follow-up by using subdistribution Cox proportional hazard. To assess the effect of aging, we also did subgroup analyses for different age interval. In the primary analysis, patients who were stratified into 4 groups on the basis of age. Since there was not same window for all the groups, we performed a sensitivity analysis with fixed interval of 10 years between 6 subgroups (age 25-34, 35-44, 45-54, 55-64, 65-74 and > 75 years) to test the robustness of our study design and results. For survival analysis, we adopted cumulative incidence function curves for incidence of ischemic stroke in HCM patients and matched controls and comparison was conducted using the Fine and Gray test [24]. Data are displayed as mean ± SD for continuous variables and as proportions for categorical variables. All of the analyses were conducted using the Statistical Package for the Social Sciences (SPSS) for Windows, Version 22.0 (SPSS, Inc., Chicago, Illinois). Values of P<0.05 were considered significant; all are reported as 2 sided.

## CONCLUSIONS

This large nationwide cohort study observed the risk of ischemic stroke in patients with HCM but without AF was comparable to those of matched general population with AF. Stratified by CHA_2_DS2-VASc scores, HCM subjects with low scores (≤2) had significantly increased risk of ischemic stroke than matched general group. In conclusion, HCM patients without AF also possess high risk of ischemic stroke and therapy of anti-coagulants may be necessary, especially those with elder age.

## Supplementary Material

Supplementary Tables
