# Supplementary material for: Risk of ischemic stroke in patients with hypertrophic cardiomyopathy in the absence of atrial fibrillation – a nationwide cohort study
Source: Aging (Albany NY). 2019 Dec 2;11(23):11347–57. doi: 10.18632/aging.102532 (PMC6932926; doi:10.18632/aging.102532)
Supplement: Supplementary Tables [file aging-11-102532-s001..pdf]

SUPPLEMENTARY TABLES

Supplementary Table 1. Predictors of ischemic stroke in patients with HCM without documented atrial fibrillation.

Predictor	Univariate analysis		Multivariate analysis	
	Crude HR (95% CI)	P value	Crude HR (95% CI)	P value
Age at initial HCM diagnosis (per 1 increase)	1.046 (1.040 – 1.053)	<0.001	1.069 (1.061 – 1.077)	<0.001
CHA2DS2-VASc	1.092 (1.044-1.142)	<0.001	1.102 (1.036 – 1.154)	<0.001
Female	1.350 (1.158-1.574)	0.003		
CKD	0.939 (0.715-1.233)	0.650		
Sudden cardiac death	1.763 (1.234 – 2.519)	0.002	2.105 (1.464-3.028)	<0.001

CI indicates confidence interval; HR, hazard ratio; HCM, hypertrophic cardiomyopathy.

Supplemental Table 2. Risk of ischemic stroke in patients with hypertrophic cardiomyopathy but in the absence of atrial fibrillation, treated the matched general population with atrial fibrillation as reference group, by using subdistribution Cox proportional hazards.

HCM w/o AF vs General population with AF	Subdistribution hazard ratio		
	General population	HCM patients	95% confidence interval
Overall	1	0.982	0.831-1.191
Age, years			
20-39	1	0.672	0.352-2.513
40-64	1	0.812	0.527-2.013
65-74	1	1.152	1.001-1.421*
≥ 75	1	1.822	1.522-2.731*

* p value < 0.05.
Abbreviations: AF, atrial fibrillation; HCM, hypertrophic cardiomyopathy.

Supplementary Table 3. Hazard ratio (95% confidence interval) of ischemic stroke in patients with hypertrophic cardiomyopathy but in the absence of atrial fibrillation, treated the matched general population with atrial fibrillation as reference group.

HCM w/o AF vs General population with AF	General population	HCM patients	95% confidence interval
Overall	1	0.963	0.853-1.092
Age, years			
25-35	1	0.592	0.251-2.113
35-45	1	0.648	0.467-1.648
45-55	1	0.742	0.497-1.529
55-65	1	0.892	0.562-1.748
65-75	1	1.278	1.070-1.335*
> 75	1	1.757	1.435-2.152*

* p value < 0.05.
